# Food allergy population thresholds: DBPCFC data for rice, kiwi, apple, peach, carrot, maize, tomato, and other less common allergenic foods

**DOI:** 10.1186/2045-7022-5-S3-O11

**Published:** 2015-03-30

**Authors:** Ben Remington, Joost Westerhout, Marty Blom, Astrid Kruizinga, Steve Taylor, Geert Houben, Joe Baumert

**Affiliations:** 1 TNO, Zeist, The Netherlands; 2 FARRP, Lincoln, NE, USA

## 

The "Big 8" food allergens consists of cow's milk, eggs, wheat, peanuts, tree nuts, soybeans, fish, and crustacean shellfish and account for ~90% of the allergic reactions to foods. During the scientific review of the VITAL^®^ (Voluntary Incidental Trace Allergen Labelling) program, individual challenge data were gathered for the Big 8 food allergens (plus mustard, sesame and celery) and, where possible, population threshold distributions were calculated. The VITAL^®^ review process yielded over 1800 individual minimal eliciting doses (MED) for these allergens. However, ~80% of the previously gathered threshold data was generated by only 4 allergens: peanut (~40%), milk (~20%), egg (~10%) and hazelnut (~10%).

Furthermore, over 150 foods have been reported to cause allergic reactions and clear data gaps still exist. For example, data gathered on celery and fish were not sufficient to generate a population distribution, threshold data for cashew were only available in children.

Minimal eliciting doses for less commonly allergenic foods provides valuable information to all food allergy stakeholders. This study examines the current publically available MED data for rice, kiwi, apple, peach, carrot, maize, tomato, and other less common allergenic foods. This study will also try to minimize the datagaps of the "Big 8" food allergens. Where possible, population based threshold distributions and ED-values are calculated. Potency data from the less common allergens are compared to previously gathered data on the Big 8 food allergens. Additionally, all data collected during this study regarding the potency of less common allergens will be incorporated into the continued development of a validated, predictive and accepted allergen risk assessment strategy for existing and new protein sources and new or modified protein (containing) products.

The results from this study should enable government and food industry risk managers to make more informed decisions regarding less commonly allergic foods.

